# Identification of a Gene Regulatory Network Necessary for the Initiation of Oligodendrocyte Differentiation

**DOI:** 10.1371/journal.pone.0018088

**Published:** 2011-04-07

**Authors:** Victoria A. Swiss, Tung Nguyen, Jason Dugas, Adiljan Ibrahim, Ben Barres, Ioannis P. Androulakis, Patrizia Casaccia

**Affiliations:** 1 Department of Neuroscience and Genetics and Genomic, Mount Sinai School of Medicine, New York, New York, United States of America; 2 BioMAPS Institute for Quantitative Biology, Rutgers University, Piscataway, New Jersey, United States of America; 3 Departments of Biomedical Engineering and Chemical Biochemical Engineering, Rutgers University, Piscataway, New Jersey, United States of America; 4 Department of Neuroscience, Stanford University School of Medicine, Stanford, California, United States of America; Emory University, United States of America

## Abstract

Differentiation of oligodendrocyte progenitor cells (OPCs) into mature oligodendrocytes requires extensive changes in gene expression, which are partly mediated by post-translational modifications of nucleosomal histones. An essential modification for oligodendrocyte differentiation is the removal of acetyl groups from lysine residues which is catalyzed by histone deacetylases (HDACs). The transcriptional targets of HDAC activity within OPCs however, have remained elusive and have been identified in this study by interrogating the oligodendrocyte transcriptome. Using a novel algorithm that allows clustering of gene transcripts according to expression kinetics and expression levels, we defined major waves of co-regulated genes. The initial overall decrease in gene expression was followed by the up-regulation of genes involved in lipid metabolism and myelination. Functional annotation of the down-regulated gene clusters identified transcripts involved in cell cycle regulation, transcription, and RNA processing. To define whether these genes were the targets of HDAC activity, we cultured rat OPCs in the presence of trichostatin A (TSA), an HDAC inhibitor previously shown to inhibit oligodendrocyte differentiation. By overlaying the defined oligodendrocyte transcriptome with the list of ‘TSA sensitive’ genes, we determined that a high percentage of ‘TSA sensitive’ genes are part of a normal program of oligodendrocyte differentiation. TSA treatment increased the expression of genes whose down-regulation occurs very early after induction of OPC differentiation, but did not affect the expression of genes with a slower kinetic. Among the increased ‘TSA sensitive’ genes we detected several transcription factors including *Id2*, *Egr1*, and *Sox11*, whose down-regulation is critical for OPC differentiation. Thus, HDAC target genes include clusters of co-regulated genes involved in transcriptional repression. These results support a de-repression model of oligodendrocyte lineage progression that relies on the concurrent down-regulation of several inhibitors of differentiation.

## Introduction

Differentiated oligodendrocytes (OLs) wrap a lipid-rich membrane, termed myelin, around axons providing insulation of electrical signals as well as trophic support [1]–[3]. As myelin formation requires the synthesis of both lipids and myelin specific proteins, such as MBP and PLP [4], a complex cellular machinery is required to properly synthesize and localize these components [5]–[8]. Therefore in addition to producing myelin proteins, the mature OL must synthesize enzymes affecting lipid metabolism and proteins involved in RNA and vesicular transport.

The differentiation of oligodendrocyte precursor cells (OPCs) into myelinating OLs requires a substantial change in the proteins which are synthesized [7], [9], [10]. This switch involves cascades of transcriptional and post-translational events mediated by DNA-binding proteins, micro-RNAs and chromatin regulators [11]–[15]. In particular, the known transcriptional mechanisms during the initial steps of OPC differentiation suggest a general de-repression model of differentiation, whereby inhibitors are repressed upon the initiation of differentiation [11].

Transcriptional changes are mediated by changes to the chromatin architecture, and a well-characterized modification occurring at the very early stages of differentiation in OPCs is the deacetylation of lysine residues on histone tails. This type of modification is required for OPC differentiation during development [16]–[19] and for efficient remyelination in disease [20]. Removal of acetyl groups from histone tails is carried out by a family of enzymes called histone deacetylases (HDACs). Inhibition of HDAC activity at the onset of oligodendrocyte differentiation prevents the morphological changes and myelin gene expression which are attributed to oligodendrocyte maturation [17].

Although it has been previously proposed that HDAC activity targets the down-regulation of transcriptional inhibitors at the onset of oligodendrocyte progenitor differentiation [19]–[21], the identification of HDAC target genes remains elusive. We have approached this issue by generating new tools to interrogate the oligodendrocyte transcriptome and comparing the kinetic profile of gene expression in differentiating OPCs in physiological conditions, with that of OPCs treated with the HDAC inhibitor trichostatin A (TSA). Although several studies have been previously published on the oligodendrocyte transcriptome during development [22]–[25] or in demyelinating disorders [26]–[28], the issue of temporal expression profiling and co-regulation of functionally related genes has not been addressed. In this manuscript we first describe a kinetic clustering algorithm to define the sequential patterns of genes expressed during OPC differentiation and then we define HDAC-target genes.

## Results

### Dynamic co-expression of genes involved in the same biological processes

**Figure 1:**
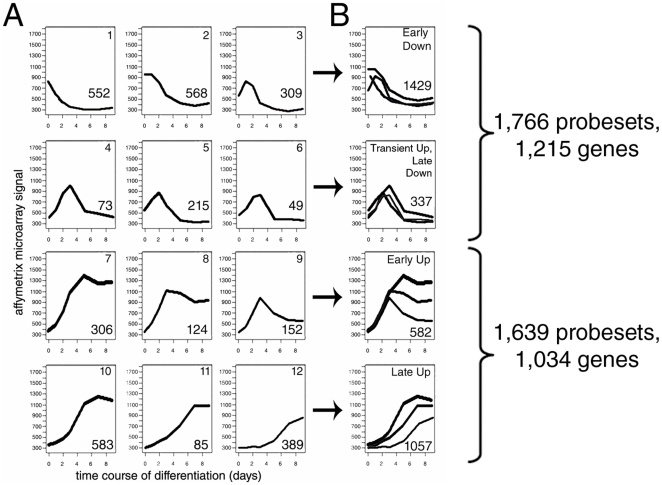
Clustering of co-expressed genes during oligodendrocyte differentiation. A. Microarray probes whose levels changed by ≥2 fold during oligodendrocyte differentiation were grouped into co-expressed clusters depending on their expression kinetics during the entire time course of differentiation. Note that the raw values were analyzed over time and they were not normalized against reference genes. Each gene cluster is represented by a graph displaying the average expression level (y-axis) over the entire course of oligodendrocyte differentiation (x-axis). Cluster ID numbers are in the upper corner of each graph and the number of probes within each cluster is found in the lower right of each graph. B. Clusters were further grouped together to define ‘early down’, ‘transient up, late down’, ‘early up’ and ‘late up’ groups. The total number of probesets and genes is listed on the right.

To understand the dynamics of global gene expression during oligodendrocyte differentiation, we analyzed a comprehensive microarray dataset which included expression values of all transcripts during a detailed time course of oligodendrocyte progenitor differentiation. This dataset was obtained from RNA samples collected at D0 (Day 0), D1, D2, D3, D5, D7, and D9 from primary OPCs that were differentiated by removal of the mitogen platelet derived growth factor (PDGF-AA) and the addition of thyroid hormone (T3) [25]. The affymetrix standard internal controls were used to normalize the signals in the microarray chip. The average probe signal for an entire microarray dataset was used to normalize the different samples. Each signal was averaged among distinct biological replicates at each time point. The analysis described here did not include any normalization to a particular set of “housekeeping genes”, but was based on the raw values. Only 3,405 probe sets (representing 2,249 annotated genes) out of 26,202 were further analyzed because they exhibited at least a two-fold expression level change during the course of differentiation. These data were subjected to a consensus clustering algorithm (K* = 7, in this case) to define the temporal profile of gene expression. This unbiased analysis of the oligodendrocyte transcriptome, revealed 12 distinct significant patterns of gene expression, each containing a certain number of transcripts. For this reason they were called “clusters” (Fig. 1A. For a full list of genes included in each cluster see Table S1). Clustering was performed by taking into account the overall temporal kinetics of gene expression throughout the differentiation process and not by comparing normalized transcript levels at each discrete time point. Genes whose expression decreased over time in differentiation conditions, were found within clusters 1–6. Transcripts in clusters 1–3 were rapidly down-regulated prior to day 3 whereas genes within clusters 4–6 were transiently upregulated and then returned to basal levels. In addition, genes within cluster 1 were down-regulated within the first day of differentiation, whereas genes in cluster 2 were down-regulated slightly afterwards, but prior to those in cluster 3. Genes whose expression increased over time in differentiation conditions were found within clusters 7–12. Genes within clusters 7–9 were rapidly up-regulated prior to day 3, while those within clusters 10–12 were characterized by a progressive increase throughout differentiation. In some cases, genes with a similar timing of expression were differentially clustered based on the transcript levels. For instance cluster 7 transcripts increased to a greater extent than those within cluster 8. The graphic representation of these clusters (as shown in figure 1) is an indication of the average kinetic expression throughout the OPC differentiation process and does not represent the sum of normalized discrete average transcript levels at each time point of differentiation. So while the graphic representation of the kinetics of genes expression incluster 6 and 9 might reveal an apparent overlap if the graph is analyzed at a given time point (i.e. the transcripts are up-regulated from day 0 to day 3), genes that belong to one clusters are uniquely represented only within that group (i.e. cluster 6 genes return to basal levels and are thereore not included in cluster 9, whose genes continue to be up-regulated throughout differentiation), based on the overall expression profile evaluated throughout the process of oligodendrocyte differentiation. To provide a simpler classification of our kinetic profiles we merged clusters into four groups: ‘early down’, ‘transient up, late down’, ‘early up’, and ‘late up’ genes based on the temporal profile of expression (Fig. 1B).

In order to understand the general functions of co-expressed genes we analyzed their gene ontology using the Database for Annotation, Visualization and Integrated Discovery (DAVID) software package [29], [30]. Each cluster of co-expressed genes was independently analyzed for enriched ontology terms found in ‘Biological process’ or ‘Cellular Compartment’ libraries. Enriched terms (*p* Value of enrichment < 10^−3^) were compared for all clusters (for all enriched ontology terms see Table S2). Strikingly, we found related ontology terms which were strictly correlated with specific co-expressed clusters. For example, terms associated with cell cycle (‘cell cycle’, ‘mitosis’, ‘M phase’, and ‘spindle’) were highly enriched within cluster 3 and were not associated with any other cluster.

**Figure 2:**
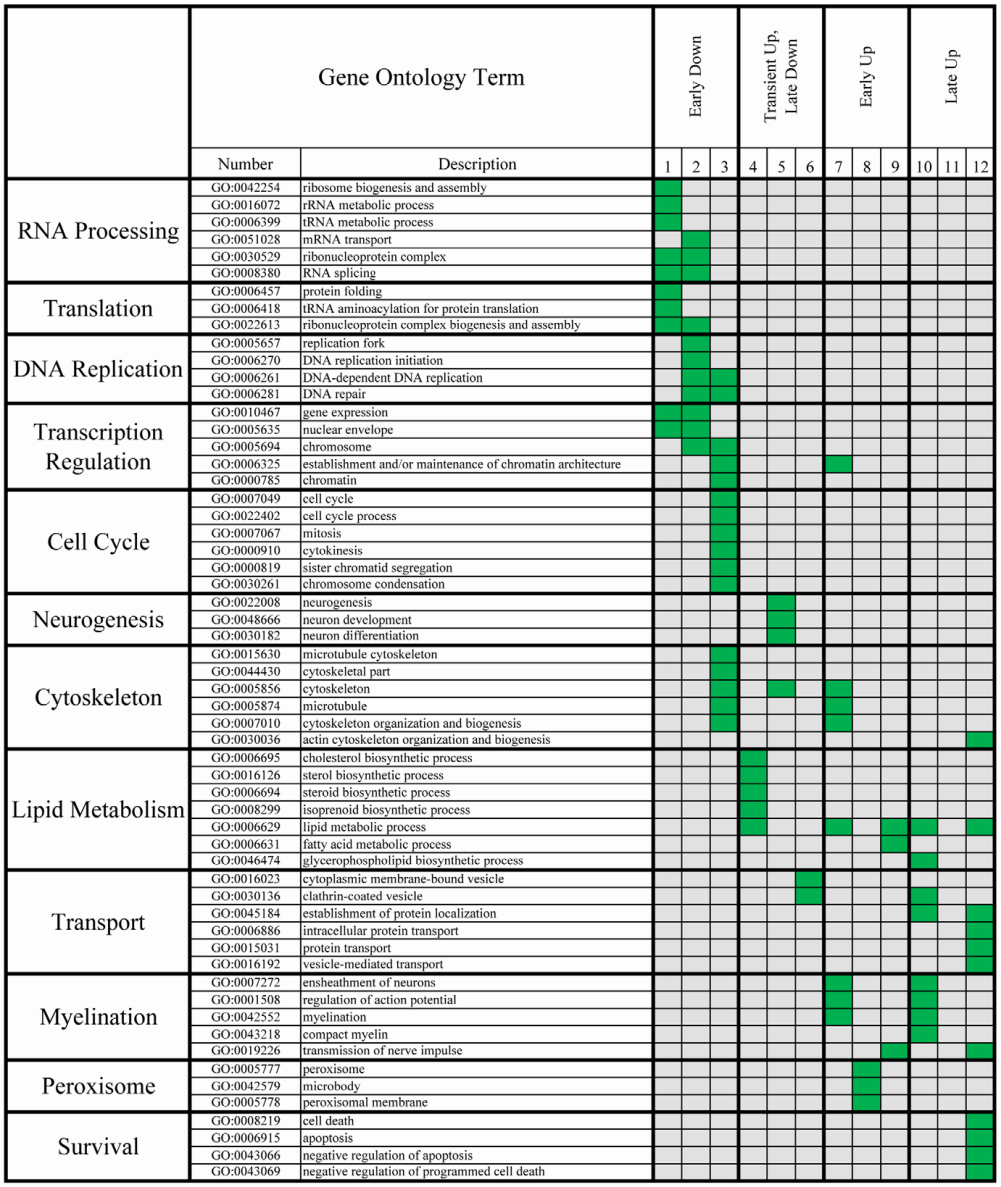
Co-expressed genes have similar biological functions. Each cluster of co-expressed genes was analyzed for ontology, as listed on the top of the chart. Non-redundant ontology terms were listed and are sub-categorized according to function. Terms with a statistically significant enrichment (p Value<10^−3^) above the background set (whole genome) are represented as a green box for each cluster.

The sequential waves of gene expression reflected the order of the biological processes occurring during the differentiation of oligodendrocyte progenitors (Fig. 2). Clusters of genes which were down-regulated during the early stages of differentiation included those involved in RNA processing, such as *Bat1a*, *Eif1a*, *Eif3S9*, *Eif4EBP1*, *Nol5a*, *Smn1* (all found within cluster1) and the heterogenous nuclear ribonucleoproteins *hnRNPA1, hnRNPA2B1, hnRNPA3, hnRPD, hnRNPH1, hnRNPR*, and *hnRNPU* (all found within cluster 2). They also included genes related to the cell cycle (e.g. *Ccna2*, *Cdc2a*, *Cdc25b, Ccnb1*, *Gmnn*, *Top2a*) and several important transcriptional regulators (e.g. *Arx, Cebpb, Egr1, Id2, Klf4, Klf10 and Sox11*). These findings are consistent with the concept that the early stages of OPCs differentiation are characterized by events modulating RNA processing [5], [31]–[33], cell cycle control [34]–[36], and down-regulation of transcriptional inhibitors of differentiation [13], [14].

The genes associated with cytoskeletal remodeling (e.g. *Ptk9, Sema6a, Sirt2*) were enriched within the first wave of increased genes (‘Early Up’), whereas genes involved in protein and vesicular transport (e.g. *Abca1*, *Rab5a*, *Rab21*, *Vamp1*) were enriched within the second wave of increased gene clusters (‘Late Up’) and this kinetic pattern underscores the functional relevance of cytoskeletal components for RNA and protein transport, myelin formation and stability [37]. Interestingly, we observed that several genes involved in lipid metabolism (e.g. *Elovl6*, *Fdft1*, *Fdps*, *Hmgcr, Lss, Scd1*) were initially up-regulated but returned to basal levels at the end of the differentiation process (cluster 4 of figure 1), while others continued to be expressed at high levels (e.g. *Acsl1*, *Dgat2*, *Pnlip, Sc5d*). Some myelin-specific transcripts (e.g. *Mbp*, *Plp*, *Ugt8*) and peroxisome-specific genes (e.g. *Abcd3*, *Cat*, *Pxmp4*, *Scp2*) were identified in ‘early up-regulated’ clusters and followed similar kinetics to the enzymes involved in lipid metabolism. The group of genes displaying a late increase during oligodendrocyte differentiation included myelin transcripts such as *Mag*, *Mobp*, and *Mog*.

Thus, the kinetic analysis of the transcriptome revealed a temporal sequence of events in which global down-regulation occurred during the very early steps of OPC differentiation (1,429 out of 1,766 total down-regulated probesets prior to day 3). This wave was followed by a later increase of gene transcripts related to lipid metabolism and myelin proteins (1,057 probesets out of 1,639 probesets that were up-regulated after day 3). OPC differentiation was associated with an overall decrease of gene expression that occurred around cell cycle exit and preceded the synthesis of lipid enzymes and myelin genes.

### Inhibition of HDAC activity halts the transcriptional program of differentiation

Previous work had suggested that gene repression at the onset of oligodendrocyte differentiation is associated with HDAC activity and is necessary for myelination [16], [17], [20]. In an effort to define target genes of HDAC activity during oligodendrocyte differentiation we performed microarray analysis on OPCs differentiated for one day in the presence or absence of the HDAC inhibitor Trichostatin A (TSA). A total of 2,592 genes (3,958 probesets) exhibited at least a two-fold difference compared to controls upon TSA treatment, and of these genes 66% were increased (1,718 genes) and 33% were decreased (874 genes) in expression.

We then asked whether the effect of HDAC inhibition was random across the genome (independent of the transcriptome), or rather selective for a subset of genes which are regulated during oligodendrocyte differentiation. Since the TSA treatment was limited to the first 24 hours, we analyzed genes within the early oligodendrocyte transcriptome (clusters 1–9, totaling 1,991 genes) for their responsiveness to TSA treatment. We found that the ‘TSA sensitive’ genes (total of 2,592 genes) included 526 genes that were significantly increased or decreased during the early stage of OPC differentiation. A Chi Square test with Yates correction revealed that this overlap of TSA affected genes (total n = 2592), and the set of genes normally changed during the early time course of oligodendrocyte differentiation (n = 1991) was not independent of one another (X^2^ = 98.1, p<0.0001). We therefore conclude that HDAC inhibition on oligodendrocyte differentiation was not the consequence of a global change in gene expression, but rather due to a specific effect on genes modulating the oligodendrocyte differentiation program. Therefore we further invested the role of HDAC target genes so we could learn more about oligodendrocyte biology.

### HDAC inhibition increases the expression of transcriptional regulators, but not cell cycle genes

**Figure 3:**
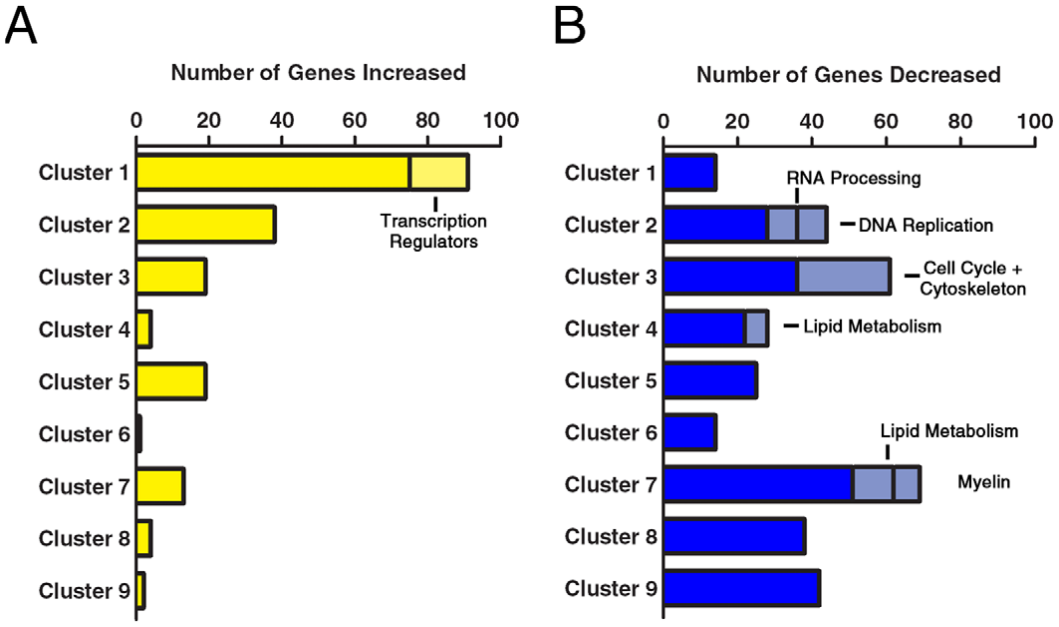
HDAC inhibition in differentiating OPCs halts the transcriptional program of differentiation. OPCs were differentiated for one day in the absence or presence of the HDAC inhibitor Trichostatin A (TSA), mRNA was extracted and analyzed by microarray analysis. TSA sensitive genes were overlapped onto the early oligodendrocyte transcriptome (clusters 1–9). A. The light yellow bar graphs represent the number of genes within each kinetically defined cluster which were increased by TSA treatment. B. The light blue bar graphs represent the number of genes which were decreased by TSA treatment. Groups of genes enriched in a particular ontology group are indicated and labeled.

We first asked whether TSA treatment similarly affected all the distinct gene clusters or whether the effect was prevalent in specific ontology groups. We observed that TSA treatment increased the expression of genes within cluster 1 (immediately down regulated genes) and comprised several transcriptional regulators (e.g. *Cebpb*, *Cited2, Egr1, Egr1, Klf4, Klf10, Lmo4, Nab2, Nolc1, Sox11, and Zcchc12*), including those that have been reported to be expressed in immature OPCs (i.e. *Egr1* and *Sox11*) [38], [39]. Overwhelmingly, genes within the other clusters were down-regulated in response to TSA and included transcripts involved in lipid metabolism, myelin transcripts and other genes previously reported to be critical for oligodendrocyte development such as *Qki* and the transcription factors *Nkx2.2 and Nkx6.2* (Fig. 3), for a partial list of genes changed upon TSA treatment see Table 1.

**Table 1:** Representative genes whose expression is significantly altered by TSA treatment.

Gene Symbol	Cluster Number	Gene Function	Fold change in expression
BEX4	1	Transcriptional Regulator	2.8
CEBPB	1	Transcriptional Regulator	3.0
CITED2	1	Transcriptional Regulator	2.3
EGR1	1	Transcriptional Regulator	4.9
EGR2	1	Transcriptional Regulator	6.7
FHL3	1	Transcriptional Regulator	2.5
KLF10	1	Transcriptional Regulator	2.5
KLF4	1	Transcriptional Regulator	3.9
LMO4	1	Transcriptional Regulator	2.4
NAB2	1	Transcriptional Regulator	4.3
NOLC	1	Transcriptional Regulator	17.8
RGD1562672	1	Transcriptional Regulator	3.7
SOX11	1	Transcriptional Regulator	2.9
ZCCHC12	1	Transcriptional Regulator	6.1
HNRPA1	2	RNA processing	−2.5
HNRPA2B1	2	RNA processing	−2.5
HNRPH1	2	RNA processing	−2.7
MCM4	2	DNA Replication	−2.7
MCM6	2	DNA Replication	−2.6
MCM7	2	DNA Replication	−2.4
ASF1B	3	Cell cycle/cyotskeleton	−2.8
BARD1	3	Cell cycle/cyotskeleton	−5.6
CDC2A	3	Cell cycle/cyotskeleton	−4.3
FOXM1	3	Cell cycle/cyotskeleton	−4.6
CCNB1	5	Cell cycle/cyotskeleton	−5.9
CCNB2	5	Cell cycle/cyotskeleton	−5.9
ACSL1	7	Lipid Metabolism	−2.1
DHCR24	7	Lipid Metabolism	−3.9
PCYT2	7	Lipid Metabolism	−2.7
PNLIP	7	Lipid Metabolism	−23.4
CD9	7	Myelination	−2.3
CNP1	7	Myelination	−2.8
MBP	7	Myelination	−2.8
PLP	7	Myelination	−3.5
MAG	10	Myelination	−20.7
MOBP	10	Myelination	−2.8
MOG	10	Myelination	−2.1

Gene symbols are listed along with their kinetic cluster number describing their normal expression during oligodendrocyte differentiation (see figure 1), the functional gene ontology groups which they are associated (see figure 2), and the expression fold change found by microarray analysis in OPCs differentiated for one day in the presence of TSA compared to those OPCs differentiated for one day without TSA.

**Figure 4:**
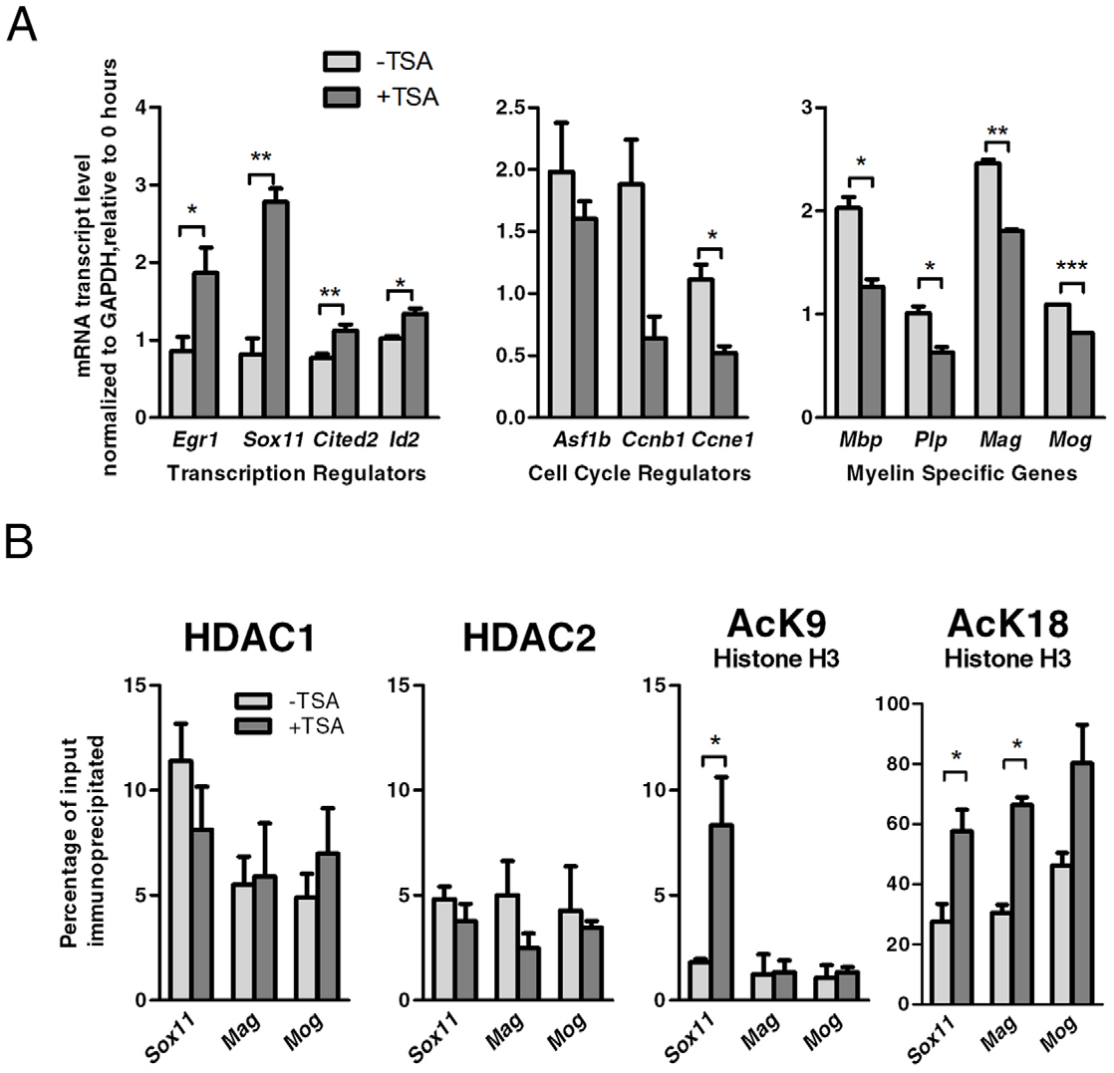
HDAC activity directly regulates the expression of Sox11. A. Quantitative RT-PCR was performed to validate changes of transcript levels for transcriptional regulators, cell cycle regulators and myelin specific genes in OPCs either untreated or treated with TSA for one day. The data reflect the results of qPCR results performed in duplicate from 2–4 independent biological replicates (* p<0.05; ** p<0.01, *** p<0.001 as determined by two-tailed t-test). B. Quantitative Chromatin Immunoprecipitation of samples collected from OPCs either untreated or treated with TSA and then immunoprecipitated with antibodies specific for HDAC1, HDAC2, acetylated lysine 9 on histone H3, and acetylated lysine 18 on histone H3. A mock immunoprecipiation was used (not shown) as a negative control. The experiment was oerformed in triplicate from two separate biological replicates. Data are represented as mean ± SEM, * p<0.05 as determined by two-tailed t-test.

The microarray data were validated by reverse transcription followed by quantitative PCR (qRT-PCR) with primers specific for transcription factors increased within cluster 1 such as *Cited2, Egr1* and *Sox11* (Fig. 4A). We further validated the decreased expression of the myelin specific genes *Mbp*, *Plp*, *Mag*, and *Mog* in response to TSA treatment by qRT-PCR (Fig. 4A). It is worth noting that the transcript levels of genes involved in RNA processing and cell cycle regulation (i.e. *Asf1b*, *Cycin B*, *Cyclin E*, *Cyclin F, Cyclin H, Cdc27, Cdc2A, CdcA1, Topo1,* and *Mki67*) were much lower in the TSA-treated samples than in controls, we further validated also the decreased expression of *Asf1b*, *Ccnb1*, and *Ccne1* (Fig. 4A). The dramatic decrease of the transcripts for cyclins and other positive regulators of proliferation was consistent with the idea that inhibition of HDAC activity impaired differentiation independently from proliferation, since the cells remained capable to exit from the cell cycle [17]. Based on this overall analysis of the oligodendrocyte transcriptome after TSA treatment, we conclude that TSA-sensitive genes are involved in a global halt of the transcriptional network of oligodendrocyte differentiation, occurring after cells have exited from the cell cyle.

We next determined whether TSA treatment of OPCs was associated with increased histone acetylation at the promoters of genes displaying increased transcript levels. We chose to focus on the analysis of the *Sox11* promoter and investigate the acetylation of lysine residues on the tail of histone H3 at position 9 (AcK9H3), because this is a critical residue that has been linked to the activation of transcription of *Egr1* [40]–[42]. By performing quantitative chromatin immunoprecipitation (qChIP) we found that histone H3 at the transcriptional start site of *Sox11* was hyperacetylated on lysine 9 residue in TSA treated cells, compared to untreated controls (Fig. 4B). In contrast, acetylation of lysine 9 at the transcriptional start sites of the myelin genes *Mag* and *Mog* remained constant, despite their decreased transcript levels after TSA treatment. Interestingly, the acetylation of lysine 18 of Histone H3 was increased by TSA in all gene promoters analyzed and did not correlate with increased gene expression. Together these data suggest a residue-specific role of acetylation, with K9 specifically involved in the regulation of transcription. Together with previous studies on *Egr1* [40]–[42], our results on the *Sox11* promoter indicate that acetylation is an important mechanism of modulation of genes involved in transcriptional repression, but not of myelin genes.

### Egr1 and Sox11 expression inhibit oligodendrocyte differentiation

**Figure 5:**
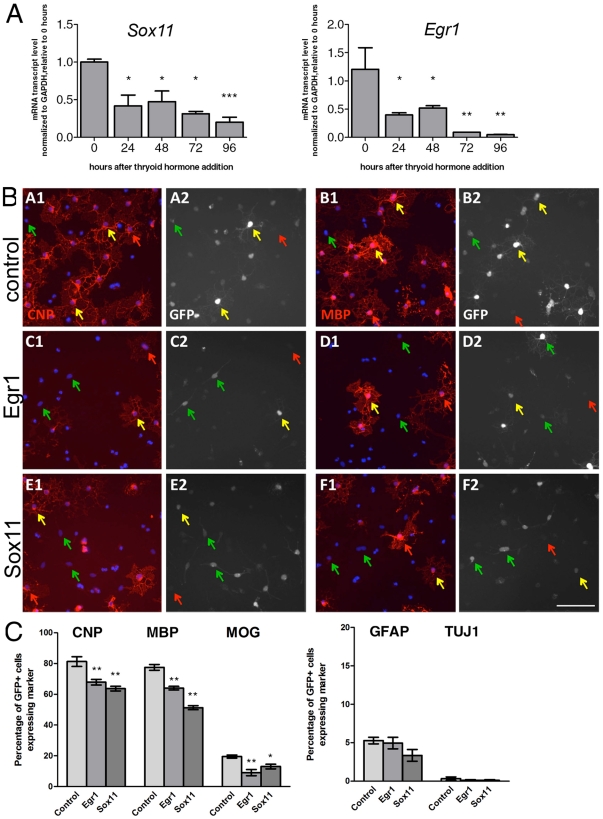
Down regulation of *Sox11* and *Egr1* is important for normal oligodendrocyte progenitor differentiation. A. Quantitative RT-PCR to measure *Sox11* and *Egr1* transcript levels in RNA samples isolated from OPCs differentiated for 0, 24, 48, 72, and 96 hours. Error bars ±S.E.M, ** p<0.001, * p<0.005 as determined by student two-tailed T-test. B. Images of purified P7 rat OPCs transfected with either pC1-eGFP alone (A-B, “control”), or co-transfected with either pSp-Egr1 (C–D) or pSp-Sox11 (E–F) expression vector. Transfected OPCs were cultured for 3 days in mitogen-free (-PDGF) medium to induce differentiation, and subsequently immunostained for GFP (white; A2-F2) plus either CNP (red; A1, C1, E1) or MBP (red; B1, D1, F1) and counterstained with DAPI (blue; A1-F1) to visualize nuclei. Yellow arrows indicate GFP^+^ transfected cells co-expressing CNP or MBP; green arrows indicate GFP^+^ cells negative for CNP or MBP expression; red arrows indicate untranfected cells expressing CNP or MBP. Scale bar  =  200 µm. C. Percentages of control (GFP-only), pSp-Egr1, and pSp-Sox11 transfected cells expressing the indicated oligodendrocyte specific markers (CNP, MBP, or MOG). Error bars ±S.E.M., n = 6–16 samples from 2–5 independent experiments, ** p<0.001, * p<0.005 post-hoc SNK test vs. control.

**Figure 6:**
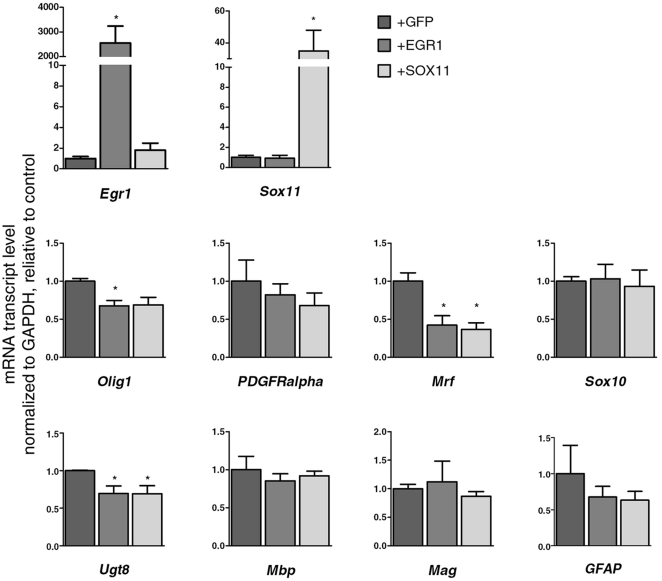
Over expression of Egr1 or Sox11 inhibits the expression of differentiation activators. Quantitative RT-PCR of RNA samples collected from OPCs cultured as decribed in figure 5 and amplified with primers specific for *Egr1* and *Sox11* to validate over-expression. The transcript levels of oligodendrocyte lineage markers (*PDGFRalpha*, *Olig1*), transcriptional activators of OPC differentiation (*Mrf*, *Sox10*), myelin genes (*Ugt8*, *Mbp*, *Mag*), and of an astrocytic gene (*Gfap*) were measured in Egr1 or Sox11 over-expressing cells and compared to GFP over-expressors. Data are represented as mean ± SEM, from 3 independent experiments (*p<0.05 as determined by two-tailed t-test).

Because several genes expressed in cluster 1 encoded for transcriptional inhibitors of oligodendrocyte differentiation we asked whether *Egr1* or *Sox11*, that were part of cluster 1 had a similar inhibitory role. We first confirmed that *Egr1* and *Sox11* were expressed in OPC, and down-regulated during differentiation (Fig. 5A). Next, we tested the consequences of retaining their expression, using over-expression followed by the induction of differentiation for 4 days (Fig. 5B). The over-expression of either *Egr1* or *Sox11* in primary OPCs did not affect the expression of astrocytic or neuronal markers on OPCs, but decreased the proportion of oligodendrocytes immunoreactive for antibodies specific for CNP, MBP and MOG (Fig. 5C). In addition, *Egr1* or *Sox11* over-expression modulated the levels of several other transcripts that characterize the progression to mature oligodendrocytes (Fig. 6). *Egr1* overexpressing cultures, for instance were characterized by levels of *Olig1*, *Mrf*, and *Ugt8* significantly lower than GFP transfected controls. Similarly, *Sox11* overexpressing cultures were characterized by lower transcript levels for *Mrf* and *Ugt8* compared to controls. Mrf is a critical transcription factor necessary for the differentiation of oligodendrocytes [43], but still very little is known about its upstream regulation. Our data identify *Egr1* and *Sox11* as potential upstream regulators of *Mrf* expression. Together, these data demonstrate a functional role of HDAC-regulated cluster 1 genes in modulating oligodendrocyte differentiation.

## Discussion

Differentiation of OPCs into myelinating OLs encompasses a complex sequence of events including cell cycle exit, RNA processing, branching, synthesis of myelin proteins and lipids and membrane wrapping around the axon [36], [44]. At a molecular level, these events are modulated by regulating the levels of transcripts through the interplay between chromatin regulators, transcription factors and microRNAs. While studies on the oligodendrocyte transcriptome have revealed the identity of many genes contributing to these processes [22]–[25], [33], [45], [46], it remains unclear whether the process is stochastic or regulated by intrinsic mechanisms of kinetic regulation of gene expression that are dependent on the activity of chromatin modulators. In an effort to understand the rules regulating global gene expression during oligodendrocyte progenitor differentiation, we designed a kinetic clustering algorithm to interrogate the oligodendrocyte transcriptome [25] and characterized clusters of co-regulated genes defining complex waves of gene expression. We classified genes on the basis of two criteria: temporal regulation and fold change, and limited our analysis to transcripts whose levels changed at least two-fold during the time course of differentiation. These parameters set a stringent threshold and defined 12 clusters of co-expressed genes. We then used ontology software to define the biological functions associated with these clusters. We found that genes involved in RNA splicing and processing, transcriptional regulation and cell cycle control were rapidly down-regulated, while those involved in lipid metabolism, cytoskeletal reorganization and myelin gene products were subsequently up-regulated and followed by a later wave of transcripts affecting vesicular transport, late myelin genes and survival. This analysis provides the first evidence of a temporally co-regulated wave of gene expression that is consistent with the subsequent modulation of cell cycle exit and RNA regulation followed by increased cytoskeletal and myelin-specific proteins, and finally lipid transport [47]. Overall we find that during the time course of OPC differentiation, genes with parallel kinetics of expression are further related to one another and contribute to similar biological functions.

The prominent down-regulation of genes at the early stages of the differentiation process was in agreement with previous studies showing that the levels of transcriptional repressors, such as Id2 and Id4 [48]–[50], Hes5 [51], [52] and Tcf7l2 [19], [21], [53] need to be down-regulated in order to allow myelin gene expression to proceed. Previous studies, including our own, had previously shown that transcriptional repression mediated by HDAC activity is necessary for oligodendrocyte differentiation, particularly during the initial stages [16]–[19], however the network of transcripts that is changed by HDAC activity has remained elusive. By comparing the results of gene-expression profiling in OPCs treated with the HDAC inhibitor TSA with the oligodendrocyte transcriptome, we identified transcription factors that act as inhibitors of oligodendrocyte differentiation. The majority of the HDAC-regulated genes included transcriptional regulators within the first cluster of rapidly down-regulated genes, but not cell cycle genes. Among cluster 1 gene targets for HDAC, we detected *Egr1* and *Sox11* as repressors of the normal program of OPC differentiation, because at high levels they suppressed the attainment of a mature phenotype. We also showed that HDAC inhibitors enhance acetylation of critical lysine residues of histone H3 at the promoter region of *Egr1* and *Sox11* and this correlates with the increased levels of these transcripts [54]. Interestingly, TSA did not significantly modulate acetylation of histones at myelin gene promoters, thereby suggesting that acetylation/deacetylation is a signal that selectively modulates the first wave of down-regulated genes, but is not involved in the modulation of later genes.

Additional genes up-regulated by TSA-treatment included several transcripts associated with alternative lineage choices, such as *NeurodD1, Pou4f1, Numb, Bace2, Hes1* and *Dlx5* for the neuronal lineage and *Bmp6* and *S100* for the astrocytic lineage. The enrichment of these genes suggests that histone deacetylation in OPCs contributes to the suppression of genes affecting alternative cell fates. The remaining gene transcripts that were increased by TSA treatment but were outside of our stringent definition of the oligodendrocyte transcriptome included genes mediating immune response (DAVID *p* value = 5*10^−23^), such as *Cathepsin S, Rab3d*, and *Toll-like receptor 2*. Interestingly, of the several hundreds of genes that were immediately down regulated upon oligodendrocyte differentiation, only one quarter were regulated by HDAC inhibition. This suggests that additional mechanisms, possibly including histone methylation and microRNA-mediated repression, are responsible for the observed decrease in transcript levels. Indeed microRNAs have recently been shown to regulate the mRNA levels of many genes which are down regulated early during oligodendrocyte differentiation, such as *PDGFRalpha, Sox6* and *Hes5* [55], [56].

Cumulatively, these data promote a model in which various OPC-expressed transcription regulators repress oligodendrocyte differentiation and maintain OPCs in an immature state. Upon initiation of differentiation, HDACs directly deacetylate lysine residues of histones occupying the promoters of these OPC-expressed repressive transcription factors. The necessary decrease in the levels of transcriptional inhibitors occurs as the OPCs differentiate and requires the concerted action of histone deacetylases and microRNAs [55], [56].

More generally, our analysis of the oligodendrocyte transcriptome has led to a detailed description of the kinetics and the biological effects of changes in transcript expression during the process of oligodendrocyte differentiation. By using this annotated transcriptome as a resource we are able to understand the gene expression expression changes we see as a result HDAC inhibition. We envision that this approach will useful for understanding the requirement of other transcriptional events during OPC differentiation, and we hope that that our annotated transcriptome will provide a resource for future studies.

## Materials and Methods

### Clustering Analysis

The expression data for the timecourse of oligodendrocyte differentiation used for the clustering analysis was previously published [25]. The averaged raw values from 4 replicates was used for the clutering analysis without further normalization.

In order to extract significant patterns of expression from high dimensional transcriptional profiling data, we utilized our previous work on consensus clustering to obtain clusters of co-expressed genes [57]. The approach starts by constructing the agreement matrix (AM). To produce the AM, a number of different clustering methods along with different metrics (Euclidean, Manhattan, and Pearson correlation [58]) were used to minimize the biases associated with individual clustering methods and/or distance metrics [59]. In our implementation, we are using hierarchical clustering (hclust), divisive analysis clustering (diana), fuzzy analysis clustering (fanny), partitioning around medoid (pam) with Pearson correlation and Manhattan metric, k-means (kmeans), fuzzy c-means (cmeans), self-organizing map (som), and model-based clustering (mclust) with Euclidean metric as the core clustering methods [60], [61]. Since clustering results are highly dependent on the initial number of clusters, the sensitivity of the AM was also examined as a function of the input number of clusters to find a ‘suggestive’ optimal number of clusters K* for the dataset [57]. Subsequently, an agglomerative hierarchical clustering algorithm is applied to cluster the data using the AM as the input similarity distance matrix (consensus clustering). The algorithm starts with every gene belonging to a cluster and then grouping two clusters into a new one at each iteration. Any two genes belonging to the new cluster need to have an agreement level greater than or equal to a user-defined agreement level δ (70% in this study). Finally, we applied a trivial-cluster removal procedure with a given p-value (0.05 as default) and obtained twelve significant patterns of expression which are shown to be critical in the dynamic transcriptional program of differentiating oligodendrocytes. Detailed discussion on the algorithm and its implementation can be found in our earlier publication [57].

### Ontology Analysis

Ontology analysis of each co-expressed group of genes was performed using the DAVID ontology software as previously described [29], [30] using the whole genome as the background dataset and an EASE score < 10^−3^ considered highly enriched ontology groups.

### Animals

Sprague-Dawley rats were purchased from Taconic. All the procedures described in this manuscript were performed to minimize distress and use of animals, in accordance to the protocol with approval number 08–676, that was reviewed and approved by the Institutional Animal Care and Use Committee (IACUC) at Mount Sinai School of Medicine.

### Cell Culture

For most experiments, OPCs were isolated from the cortex of postnatal day 1 rats, and cultured according to McCarthy and de Vellis [62]. After being shaken from the flasks, OPCs were labeled with the A2B5 antibody and further purified using magnetic beads (Miltenyi Biotec, Auburn, CA). Cells were maintained proliferating by the addition of PDGF (10 ng/ml) in ODM medium (DMEM, 100 µg/ml albumin, 100 µg/ml apo-transferrin, 16 µg/ml putrescine, 0.06 ng/ml progesterone, 40 ng/ml selenium, 5 µg/ml insulin, 1 mM sodium pyruvate, 2 mM l-glutamine, 100 units/ml penicillin, 100 µg/ml streptomycin). The removal of mitogens from the medium (mitogen withdrawal) was considered as the start of differentiation. This procedure led to a 95% pure population of A2B5+ cells. Once the cells were differentiated in chemically define medium, 90–95% of the cells expressed myelin and GalC by 5 days of differentiation. Treatment with trichostatin A (TSA, 30 nmol), was initiated when the culture medium was replaced with mitogen-free chemically defined ODM medium. The microarray data form Dugas et al. where obtained from cultures containing >95% OL-lineage cells. Upon differentiation, these cultures contained 90–95% GalC^+^/MBP^+^ OLs. Less than 1% of the cells in the culture expressed the neuronal marker Neurofilament-H and the remaining 5% were astrocytes.

### Microarray

For TSA treated and nontreated samples microarray analysis was processed in the Microarray Shared Research Facility at MSSM. Briefly, 50 ng of total RNA was reverse transcribed using T7-poly(dT) primer and converted into double-stranded cDNA. The cDNA was used as a template for subsequent in vitro transcription with biotin-labeled UTP at 37oC for 16 h using Genechip 3^1^ IVT express kit (Affymetrix). The resulting biotin-labeled cRNA was chemically fragmented, made into hybridization cocktail and hybridized to the Mouse Genome 430 Plus 2.0 arrays (Affymetrix) according to the Affymetrix GeneChip protocol. The array images were generated through a high-resolution GeneChip Scanner 3000 7G (Affymetrix), then converted to digitized data based on MAS 5.0 within the GeneChip Operating Software (GCOS). The data were normalized by scaling so that all chips had an average signal intensity of 150.

Data were deposited into ArrayExpress with accession no. MEXP-3028. All the data is MIAME compliant, as detailed on the MGED Society website http://www.mged.org/Workgroups/MIAME/miame.html.

### Cell transfection and immunostaining

For transfection experiments, OPCs were purified from P7 Sprague-Dawley rat brains as described previously [25] and purified by a negative selection on Ran-2 and GalC-coated immunopanning plates followed by a positive selection on O4-antibody coated immunopanning plates. All the procedures were in accordance to the IACUC Institutional committee (protocol 08-676). OPCs were plated into poly-D-lysine (pDL) coated tissue culture plastic flasks cultured in previously-described DMEM-Sato based, serum-free medium which contained 10 ng/ml PDGF-AA + 1 ng/ml NTF3 [25] for 6–7 days in vitro (DIV). OPCs were then enzymatically lifted and transfected as described previously [25], using Lonza/Amaxa (Gaithersburg, MD) OPC nucleofector kit; 2–3×10^6^ rat OPCs per transfection. Transfections were performed with 3.5 ug pC1-eGFP (control) or 1.5 ug pC1-eGFP + 2.5 ug pSp-Egr1/pSp-Sox11. Following transfection, OPCs were cultured on poly-D-lysine (pDL) coated glass coverslips in DMEM-Sato based medium lacking PDGF and NTF3 for 3 (CNP and MBP stained) or 4 (MOG staining) DIV. All cultures were maintained at 37°C in 10% CO_2_. Immunostaining of OPC and oligodendrocyte cultures for 2′,3′-cyclic nucleotide 3′ phosphodiesterase (CNP), Myelin basic protein (MBP), Myelin oligodendrocyte glycoprotein (MOG), and Green fluorescent protein (GFP) expression was performed as described previously [25]. GFAP (1∶1000) and TuJ1 (1∶500) were used to detect astrocytic and neuronal markers respectively.

### Plasmids

pC1-eGFP (Clontech, Mountain View, CA, 6084-1) was used to mark transfected cells with CMV-promoter driven eGFP production. pSPORT6-Egr1 was ordered from OpenBiosystems (Huntsville, AL) (EMM1002-4), in which a full-length EST clone of the human Egr1 cDNA is driven by a CMV promoter (EST clone was sequence-verified to contain the full-length Egr1 coding sequence). pSPORT6-Sox11 is the mouse full-length verified cDNA (OpenBiosystems MMM1013-9335073) cloned into the EcoR1 (5′) and NotI (3′) sites in the pSPORT6 vector (Egr1 excised, Sox11 inserted).

### Quantitative RT-PCR

RNA samples were isolated using Trizol® Reagent following manufacturer's instructions and cleaned using the RNeasy Mini kit (Qiagen, Hilden, Germany). The concentration of RNA was quantified using a nanodrop spectrophotometer. 0.5 µg of total RNA was used for a 20 ul reverse transcription (RT) reaction, using the SuperScript RT-PCR kit (Invitrogen). Quantitative RT-PCR was performed using Stratagene SYBR Green PCR master mix in an Applied Biosystems real-time PCR machine with primers to detect select mRNA targets. The melting curve of each sample was measured to ensure the specificity of the products and samples with an unexpected melting curve were excluded from further analysis. Data were normalized to the internal control *Gapdh* and analyzed using Pfaffl ΔΔCt method [63].

### Primers detecting RAT gene products used for TSA studies

See Table 2.

**Table 2:** Primers detecting RAT gene products used for TSA studies.

mRNA target name	Forward primer	Reverse primer
*Asf1b*	GGA CGC CGT GGG TGT GAC TG	CCG AAG CTC CGG GTC TGG GT
*Ccnb1*	TGT GGA GCA GCA TAC TTT GG	CTC CGT GTG GGA CAG GTA GT
*Ccne1*	ATG TCC AAG TGG CCT ACG TC	TCT GCA TCA ACT CCA ACG AG
*Cited2*	TCT TGG CTG CAT GAA CTT TG	CAC TGA CGA CAT TCC ACA CC
*Egr1*	TGC ACC CAC CTT TCC TAC TC	AGG TCT CCC TGT TGT TGT GG
*Gapdh*	AGA CAG CCG CAT CTT CTT GT	CTT GCC GTG GGT AGA GTC AT
*Id2*	ACA AGA AGG TGA CCA AGA TGG AA	GCG ATC TGC AGG TCC AAG AT
*Mag*	GCT GGG AGG GAA ATA CTA TTT CC	GAC GCT GTG CTC TGA GAA GGT
*Mbp*	CTC CCA GCT TAA AGA TTT TGG AAA	AAA TCG GCT CAC AAG GGA TTC
*Mog*	GAG GGA CAG AAG AAC CCA CA	CAG TTC TCG ACC CTT GCT TC
*Plp*	GCA AGG ATC TTT CAC CCT TAG AAA	TGG CTG AGT TAG GGC TTA AAT AGT C
*Sox11*	TCA TGT TCG ACC TGA GCT TG	TAG TCG GGG AAC TCG AAG TG

### Primers detecting MOUSE gene products used for overexpression studies

See Table 3.

**Table 3:** Primers detecting MOUSE gene products used for overexpression studies.

mRNA target name	Forward primer	Reverse primer
*Egr1*	GCCTCGTGAGCATGACCAAT	GCAGAGGAAGACGATGAAGCA
*Gapdh*	ACCCAGAAGACTGTGGATGG	CACATTGGGGGTAGGAACAC
*Gfap*	GCCACCAGTAACATGCAAGA	CGGCGATAGTCGTTAGCTTC
*Mag*	GGTGTTGAGGGAGGCAGTTG	CGTTCTCTGCTAGGCAAGCA
*Mbp*	AAATCGGCTCACAAGGGATTC	CTCCCAGCTTAAAGATTTTGGAAA
*Mrf*	TGGCAACTTCACCTACCACA	GTGGAACCTCTGCAAAAAGC
*Olig1*	CGACGCCAAAGAGGAACAG	GCCAAGTTCAGGTCCTGCAT
*Pdgra*	TTGGTGCTGTTGGTGATTGT	TCCCATCTGGAGTCGTAAGG
*Sox10*	GGAGATCAGCCACGAGGTAATG	GTTGGGTGGCAGGTATTGGT
*Sox11*	TCCAGGTCCTTATCCACCAG	GACGACCTCATGTTCGACCT
*Ugt8*	TGGTTGACATACTGGATCACTATACT	CGATCACAAATCCACACATATCATT

### Quantitative Chromatin Immunoprecipitation Assay (qChIP)

Protocol was adapted from the Q^2^ChIP assay as described [64]. Rat OPCs (4×10^6^) were crosslinked in 1% formaldehyde, lysed in nuclear lysis buffer (50 mM Tris-HCL (ph 8.0), 10 mM EDTA, 1% SDS, Protease Inhibitors (Roche) and PMSF) and sonicated using a Bioruptor (Diagenode) sonicator to produce chromatin with an average length of 250–500 base pairs. Chromatin was aliquoted and immunoprecipitated using protein A magnetic beads (Dynabeads- Invitrogen 100.01D) coated with 2 ug of antibody. Histone H3-AcK9 – abcam Ab1191, HDAC1 antibody – Upstate PA1-860, HDAC2 – abcam ab7029. A mock immunoprecipitation was set-up as control (No antibody). Immunoprecipitations were carried out overnight. Following immunoprecipitation, beads were washed 4 times with wash buffer (10 mM Tris-HCl(ph 7.5), 1 mM EDTA, 1 mM EGTA, 1% Triton X-100, 0.1% SDS, 0.1% Na-deoxycholate, 140 mM NaCl) and 2 times with TE buffer (10 mM Tris-HCl (ph 8.0), 10 mM EDTA). Immunoprecipitated chromatin and input DNA were reverse crosslinked in elution buffer (20 mM Tris-HCl (ph 7.5), 5 mM EDTA, 50 mM NaCl, 1% SDS) with the addition of proteinase K (50 ug/ml) by heating (68°C) and shaking (1300RPM) using a thermomixer (Eppendorf) for four hours. DNA was purified from the elution using phenol-chloroform followed by an overnight ethanol precipitation at −20°C. DNA was eluted in 200 ul of TE buffer.

Quantitative PCR was performed using primers to detect specific sites of rat gene promoters. *Sox11* F-5′ TCT TGG ACC ACA CCA TGA AG, R- 5′ GAA GCC GAG AGC AAC CTG, *Mag* F-5′ CTC CTC CCC TTC CTC CAT TAA and R- 5′GACAACAGGTTCCACCTTTCAAC *Mog* F-5′ AAT ATC TGG CAA GGG TGA CG, R-5′ CAG GAT CAG GCC AAG CTA AG. Sonicated chromatin from an unrelated sample was used to determine primer efficiency and as a reference for amount of DNA in each sample. The amount of immunoprecipitated DNA was quantified relative to the amount of the input DNA for each sample.

## Supporting Information

Table S1 **Full list of probes and gene symbols assigned to each cluster.** Included in the table are the probe numbers and genes names of those transcripts included in our clustering analysis. For each gene we provide the overall characterizartion of the kinetic profile (‘Early Down’, ‘Transient Up-Late Down’, ‘Early Up’ and ‘Late Up’), the cluster number (1–12) and the raw values from the microarray dataset from Dugas et al, 2006.
(XLS)

Table S2 **Full ontology search data separated by cluster number.** Complete summary table of all ontology terms classifying clusters 1–12 and with a *p* Value<10^−2^. Within each row, we list the “Go term”, the number and the *p* Value for each cluster (1–12) of co-expressed genes during oligodendrocyte differentiation.
(XLS)
